# A detailed land use/land cover map for the European Alps macro region

**DOI:** 10.1038/s41597-023-02344-3

**Published:** 2023-07-19

**Authors:** Thomas Marsoner, Heidi Simion, Valentina Giombini, Lukas Egarter Vigl, Sebastian Candiago

**Affiliations:** 1 Institute for Alpine Environment, Eurac Research, Viale Druso 1, 39100 Bozen/Bolzano, Italy; 2 Department of Ecology, University of Innsbruck, Sternwartestrasse 15, 6020 Innsbruck, Austria; 3 Ca’ Foscari University of Venice, Department of Economics, S. Giobbe 873, 30121 Venezia, Italy

**Keywords:** Environmental impact, Ecological modelling

## Abstract

Spatially and thematically detailed land use maps are of special importance to study and manage populated mountain regions. Due to the complex terrain, high elevational gradients as well as differences in land demand, these regions are characterized by a high density of different land uses that form heterogeneous landscapes. Here, we present a new highly detailed land use/landcover map for the areas included in the European Strategy for the Alpine Region. The map has a spatial resolution of up to 5 m and a temporal extent from 2015 to 2020. It was created by aggregating 15 high-resolution layers resulting in 65 land use/cover classes. The overall map accuracy was assessed at 88.8%. The large number of land use classes and the high spatial resolution allow an easy customization of the map for research and management purposes, making it useable by a broad audience for various applications. Our map shows that by combining theme specific “high-resolution” land use products to build a comprehensive land use/land cover map, a high thematic and spatial detail can be achieved.

## Background & Summary

Land use/land cover (LULC) maps present information on the physical land types that characterize the surface of the earth (i.e., land cover) and describe how humans use this land (i.e., land use)^1^. These maps allow to monitor land cover changes and land allocation for agriculture, urban development, nature conservation *et cetera*, and to assess the provision of ecosystem services and habitats^2,3^. The use of high resolution LULC maps is particularly important in those areas that are characterized by complex landscapes and unique geo-topographic conditions, such as mountain ranges. These areas face multiple challenges, such as biodiversity loss, a high vulnerability to climate change, and negative demographic trends, and are therefore in need of accurate and updated LULC information for their effective management^4–6^.

The European Alps represent a unique environment characterized by a great variety of ecosystems and landscapes that are increasingly threatened by different pressures^7^. Land use intensification in the valley bottoms is affecting the presence of green infrastructure elements such as hedgerows and riparian areas, leading to the isolation of natural habitats and a decrease in ecological connectivity^8^. The increase in temperatures caused by climate change is progressively opening to agriculture new areas at higher elevations, causing the upward shift of economically valuable crops^9^ as well as a natural shift in habitats^10^. Rural abandonment is causing the progressive marginalization of large areas, while urban areas are experiencing intensive urbanization with a significantly growing number of inhabitants^11^. To tackle these challenges, it is important to develop specific tools and data that inform policymaking, research, land planning and resource management^2^.

The availability of LULC maps of the European Alps that have both, a high thematic and spatial detail (i.e., maps characterized by a high spatial resolution and many LULC classes) is, however, limited. Indeed, even if the increased accessibility of “high-resolution” satellite imagery, of powerful computing capabilities, and of new computing techniques (e.g., deep learning) has brought new opportunities for the automated mapping of land cover^3^, LULC maps of the Alps still usually only fulfill one of the two desired characteristics. An example of a thematically very detailed LULC map is the Corine Land Cover map (CLC^12^ that includes 44 LULC classes^13^. However, from the spatial point of view, CLC has only a medium resolution (100 m, with a minimum mapping unit (MMU) of 25 ha), which limits its usability in mountain areas. Conversely, the map recently developed by Malinowski *et al*. 2020 has a high spatial resolution (10 m) but only 13 LULC classes^14^. The same holds true for other recent LULC maps that include the European Alps^15–17^. To improve both the spatial and the thematic detail of existent LULC maps, various methodologies have been developed by researchers: Rosina *et al*.^18^, for example, used a CLC refinement approach by integrating multiple datasets with higher spatial resolution and decreased the MMU from 25 to 1 ha, Pigaiani & Batista e Silva 2021^19^ applied a similar methodology increasing the spatial resolution to 50 m. Using similar procedures many other LULC maps have been produced, mostly focusing at the national and subnational level^20–23^. However, there has been no attempt to create a specific LULC map focused on the entire Alps with both a high spatial and thematic resolution.

Here, we present the first spatially and thematically highly detailed LULC map for the European Alps. We collected, harmonized and combined freely available datasets from 11 different sources to build a high-resolution map that includes 65 different LULC classes. By including small LULC features, this map is intended to support a wide range of analyses spanning from research to land management and decision making. For example, the spatial impact of linear elements such as roads, rivers and hedges can be analyzed and included in ecological connectivity mapping models or ecosystem service assessments. Local administrations can also benefit from the high resolution of the map, which can support landscape planning and resource-efficient management.

## Methods

**Fig. 1:**
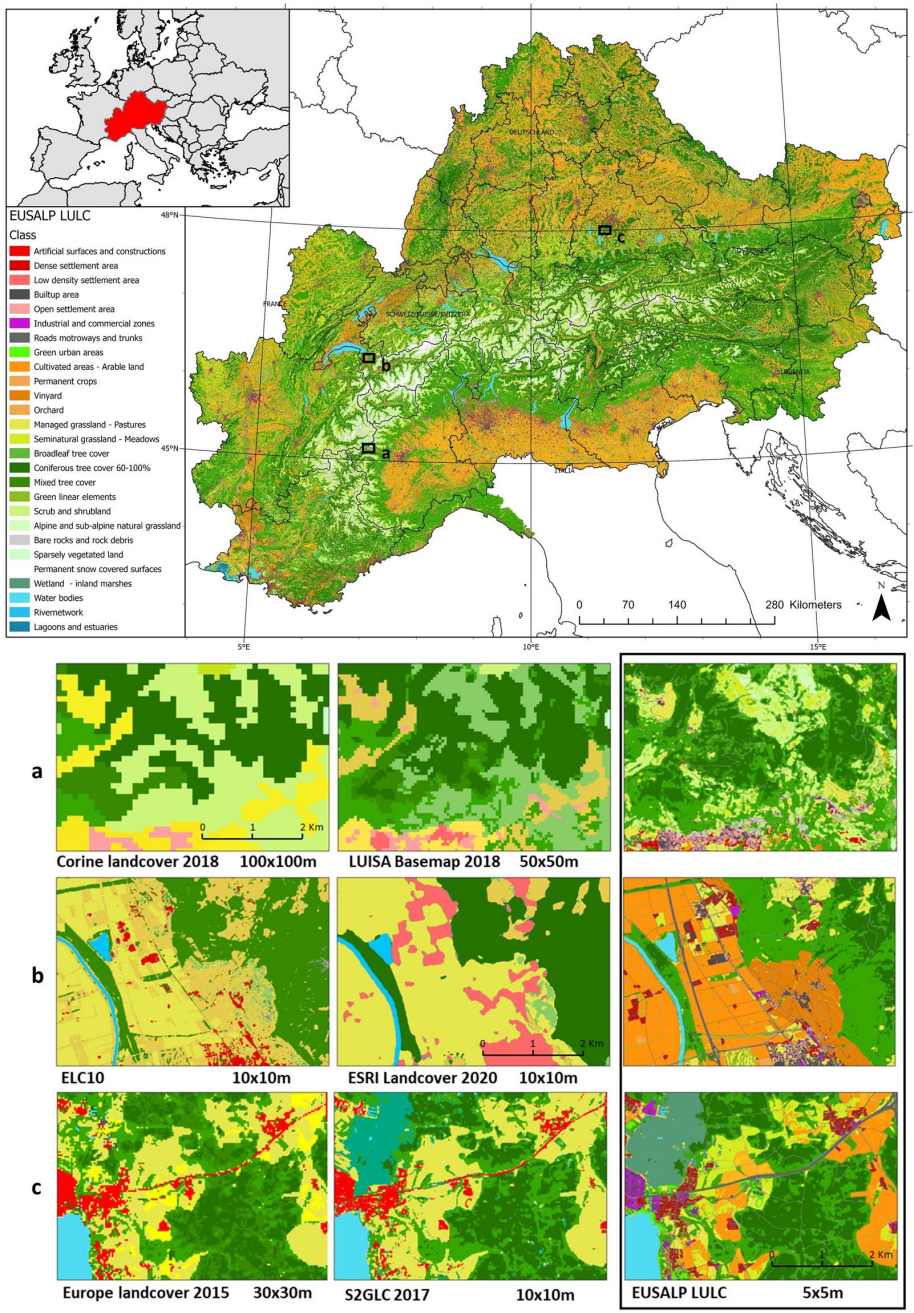
The EUSALP LULC map. The 65 LULC classes of the map aggregated into 27 classes to simplify the reading of the map. (**a**–**c**) Zoom windows showing the high resolution of the EUSALP LULC map (on the right) in comparison with other LULC products^12,14–16,19,30^.

As a reference to define the extent of the European Alps we used the area included in the European Strategy for the Alpine Region (EUSALP). This area covers a total surface of more than 440,000 km², including 7 nations and 48 administrative regions (Fig. 1).

**Fig. 2:**
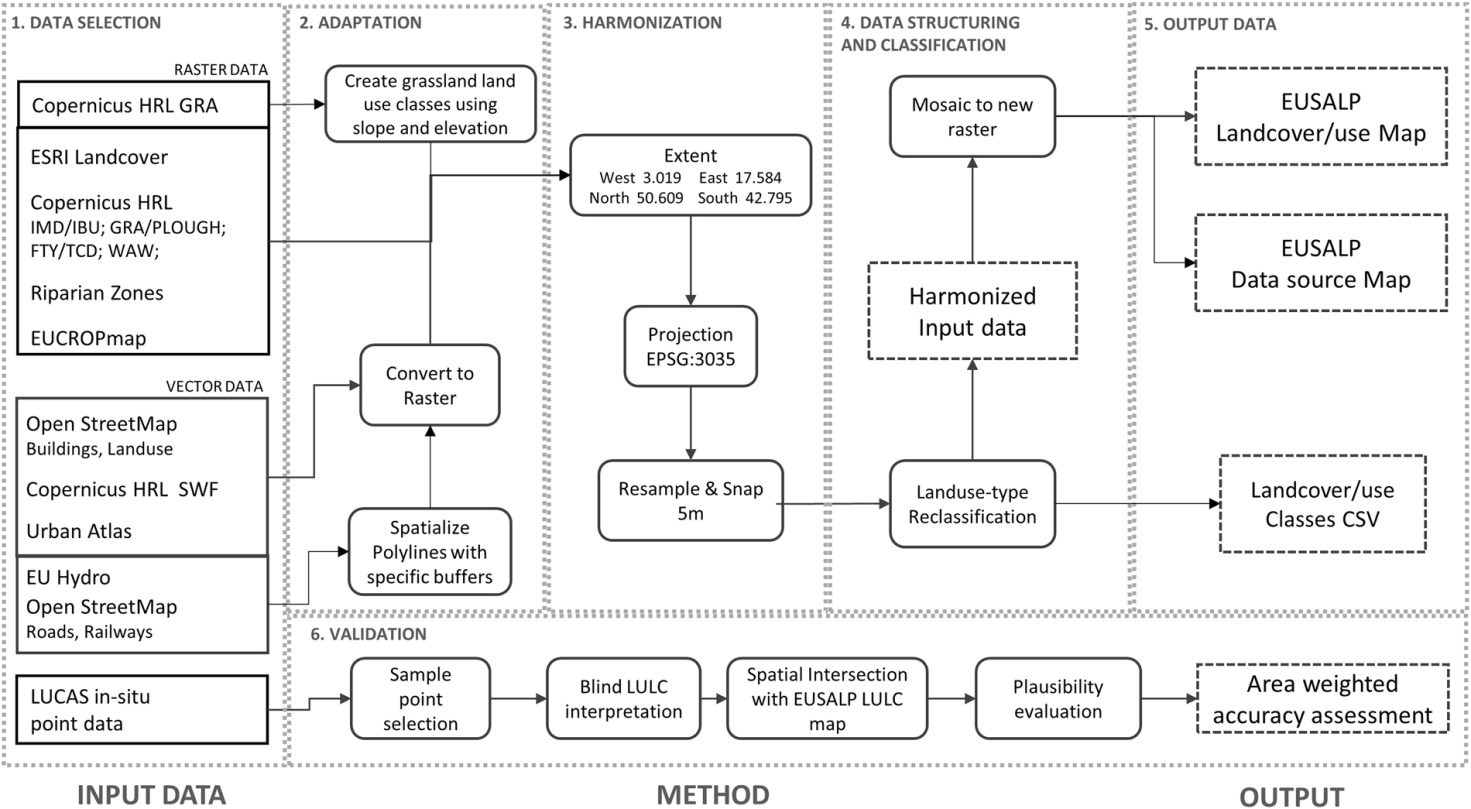
conceptual representation of the workflow used to build the EUSALP LULC map. The main steps are: (1) data selection, (2) data adaptation, (3) harmonization and (4) data structuring and classification, 5) output data and 6) validation.

The creation of the EUSALP map included the following main steps: firstly, we selected freely available datasets that covered our area of interest. Secondly, we adapted the retrieved datasets with minor alterations in order to combine high-resolution datasets from different sources. Thirdly, we harmonized all the layers using the same spatial reference system and resolution. As a last step we mosaicked the layers using a specific hierarchy based on codes given to each LULC class (Fig. 2). Finally, we validated the resulting map using an area-weighted confusion matrix approach.

### Data selection

In the first step, we collected all openly available LULC datasets that cover the whole EUSALP macro region. The following collection criteria were applied: a reference year between 2015 and 2020, a thematic accuracy higher than 80%, and a high spatial resolution (10 m). The selected data are presented in Table 1 (the area covered by the single datasets is shown in Figure S1).

**Table 1:** LULC datasets used to build the EUSALP map.

Source	Reference year	Spatial resolution	MMU	Geometric accuracy (positioning scale)	Thematic accuracy
ESRI Land Cover Map^30^	2020	10 m	Pixel based	N/A	Minimum 85% Overall Accuracy (OA)
Imperviousness high resolution layer (HRL)^36,37^	2018	10 m	Pixel based	<5 m	Minimum 90% UA/PA
Grassland HRL^38^	2018	10 m	Pixel based	<5 m	Minimum 85% OA per biogeographic region
Forest HRL^39,40^	2018	10 m	Pixel based	<5 m	Minimum 90% UA/PA
Water and Wetness HRL^41^	2018	10 m	Pixel based	<5 m	Minimum 80–85% OA
EUCROPMAP^42^	2018	10 m	Pixel based	N/A	Minimum 75–80%
*OpenStreetMap (OSM)^43^	N/A	Vector	N/A	<5 m^44^	N/A^45^
Urban Atlas 2018^46^	2018	Vector	0.25 ha	According to geo-location accuracy of satellite imagery	Minimum 80% OA
Small Woody Features HRL^47^	2015	Vector	0.02 ha	According to ortho-rectified satellite image	Minimum 80% OA
Landuse Riparian Zone^48^ & Green linear elements^49^	2018	Vector	0.5 ha	<5 m	Minimum 85% OA
**EU Hydro - Rivers and Inland water^50^	2012	Vector	1 ha	N/A^51^	N/A^51^

*see full list of selected OSM keys in Table S1.

**dataset outside of target reference range – but still the most recent dataset on this land use type.

### Data adaptation

For certain data layers (i.e., OSM Roads & Railways, EU Hydro, HRL Grassland) some adaptations were necessary prior to harmonization. Linear features (i.e., roads, railways) from the OSM were converted into polygon features by assigning the width defined by the OSM specifications (6 m width for secondary and tertiary roads as well as tracks and field roads, 10 m width for primary roads and railways, 20 m width for motorways and trunks), all tunnels were excluded. The EU Hydro River polylines were converted into polygon features using a width according to the Strahler Stream Order^24^. To characterize the use intensity of grasslands, that in the HRL Grassland dataset^25^ are defined using only a binary grassland/non-grassland classification, we divided them into three LULC classes based on elevation and slope. The classification was based on the following criteria: managed grassland (<2000m elevation and <26° slope), seminatural grassland (<2000 m elevation and >26° slope), Alpine natural grassland (>2000 m elevation)^26–28^. For the calculation we used the European Digital Elevation Model (EU-DEM), version 1.1^29^.

### Harmonization

We harmonized all the layers using the same reference system and resolution to ensure the geographical consistency of the final dataset. We projected the selected raster datasets into the same spatial reference system (EPSG:3035 ETRS89/ETRS-LAEA) and then resampled them to a resolution of 5 m using the nearest neighbor algorithm to ensure that the original pixel values are preserved, and no interpolated values are created. We also projected the vector-based datasets to EPSG:3035 and rasterized them at 5 m resolution. Next, we snapped all the layers to the same reference raster layer to ensure cell alignment. *Resolution***:** We did not perform resampling to improve the resolution of the input data, but to allow an increase in the thematic detail so that landscape features smaller than 100 m^2^ and 10 m width (e.g., buildings, roads, hedgerows, small streams) can be represented on the final map. Therefore, only in and near buildings, roads and linear elements, a map resolution of 5 m can be expected (which corresponds to approximately 15–20% of the map area).

### Data structuring and classification

We used the ESRI Land Cover Map 2020^30^ as a base layer to build our LULC map, as it is the only selected land cover dataset with complete geographical coverage for the whole research area. We added land use information to this dataset using the data presented in Table 1. To combine the layers, we first assigned specific codes to each LULC class value in all datasets (Table 2). Reoccurring LULC types across different datasets were assigned the same code (since MMU is very small and mostly pixel-based no further harmonization steps of land use types were necessary). We then overlayed the data by applying a specific layer hierarchy (Table 3) following a decision tree based on data accuracy (i.e., level of thematic and spatial detail). By assigning the value of the highest-ranking layer, we could decide which information to show on the final map, to control the uncertainties built in specific layers (e.g., presence of green linear elements in cultivated areas and grassland) and to include small LULC features (e.g. roads, single buildings, small streams in forests or grassland). All the work was done using ArcGIS Desktop 10.8.

**Table 2:** Area and brief description of the 65 LULC classes of the EUSALP map.

EUSALP Map	LULC Label	Area (ha)
11000	Artificial surfaces and constructions	322550
11100	Dense settlement area (>30%)	462599
11200	Low density settlement area (<30%)	172497
11300	Built-up area	764226
11400	Open settlement area	256910
12100	Industrial and commercial zones	352299
12210	Roads motorways and trunks	65384
12220	Road Networks	213144
12221	Roads tertiary and others	555848
12230	Railways train tracks	62112
12240	Unpaved Roads and Tracks	579138
14100	Green urban areas	88807
21000	Cultivated areas - Arable Land - Annual Crops	2305445
21211	Common wheat	2018725
21212	Durum wheat	14616
21213	Barley	594723
21214	Rye	23923
21215	Oats	1141
21216	Maize	2763510
21217	Rice	1954
21218	Triticale	809
21219	Other cereals	60
21221	Potatoes	42348
21222	Sugar beet	184238
21223	Other root crops	430
21230	Other non-permanent industrial crops	3336
21231	Sunflower	136154
21232	Rape and turnip rape	312457
21233	Soya	33055
21240	Dry pulses	59483
21250	Fodder crops (cereals and leguminous)	92470
21290	Bare arable land	35500
22000	Permanent Crops	47379
22100	Vineyard	311651
22200	Orchard	219116
23100	Managed Grassland - Pastures -	3476228
23200	Seminatural Grassland - Meadows	2793968
31100	Broadleaf tree cover	83701
31102	Broadleaf tree cover 30–60%	1335315
31103	Broadleaf tree cover 60–100%	7907733
31200	Coniferous tree cover	56645
31202	Coniferous tree cover 30–60%	842492
31203	Coniferous tree cover 60–100%	7752945
31300	Tree Cover	985801
31400	Tree cover in agricultural context	347073
31450	Tree cover in urban context	177623
31500	Green linear elements - linear woody features	469679
31600	Patchy woody features	18742
31610	Additional woody features	149203
32000	Scrub and shrubland	2007252
32100	Alpine and sub-alpine natural grassland	818190
32200	Moors and heathland - other scrubland	15375
32300	Sclerophyllous vegetation	4508
33100	Beaches, dunes, sands	32914
33200	Bare rocks and rock debris	524938
33300	Sparsely vegetated land	45662
33500	Permanent snow-covered surfaces	463822
41000	Wetland (permanent wet areas) - inland marshes	103541
41200	Peatbogs	177
42100	Coastal salt marshes	12216
42200	Intertidal flats	895
51000	Water bodies	643055
51100	River network	52971
51200	Riverbed >10 m width	6740
52100	Lagoons and estuaries	12173

**Table 3:** Hierarchy used for combining the different layers and assigning LULC classes values.

Stable	Layer name	Datasource code
1	ESRI Land Cover	1
2	Imperviousness HRL - IMD	2
3	OSM Built-up delineation	3
4	EU Hydro - Rivers	4
5	Imperviousness HRL - IBU	2
6	Urban Atlas 2018	5
7	Riparian Zones LU-LC	6
8	EU Hydro - Lakes	4
9	Grassland HRL - PLOUGH	7
10	Grassland HRL - GRA	7
11	OSM Landuse	3
12	EUCROPMAP	12
13	Small Woody Features HRL	10
14	Riparian Zones GLE	11
15	Forest HRL	8
16	Water and Wetness HRL	9
17	OSM Buildings	3
18	OSM Railways	3
19	OSM Roads	3

In case of overlap, the layer with the highest hierarchy value would be shown on the final map. (1-lowest hierarchy value, 19- highest hierarchy value).

## Data Records

We present an easily accessible and freely available high resolution LULC map of the EUSALP region that can be used to support researchers and practitioners in the field of landscape planning and management. The data is freely available through the Figshare data publisher^31^.

It includes two raster geospatial files that contain the EUSALP high resolution LULC map and a reference to the source dataset used to define each of the pixel values. The file has a pre-built color palette included to classify the 65 classes of the LULC map. The files included are:

1. EUSALP_LULC_05 m_2020.tif: a .tiff file that includes the classification of the EUSALP area based on 65 LULC classes.
2. EUSALP_LULC_data_sources.tif: a .tiff file that includes the reference information about the dataset used to define each pixel of the map (dataset name, publication year, reference year).
3. EUSALP_LULC_classes.csv: a .csv file that includes the code and description of the 65 classes of the LULC map.

## Technical Validation

The primary purpose of the present validation procedure is not to assess the individual LULC classes, but to ensure that the harmonization steps and hierarchy in combining the data are still capable of producing accurate LULC information, given that the map is built upon already validated and published input data. For more details on the validation and accuracy of the input data, see Table S2.

The assessment of thematic accuracy was carried out following the procedure applied for validation of similar LULC products^32,33^.

We applied a stratified random sampling design using the Eurostat LUCAS 2018 survey data points as the reference dataset^34^. In total, 32,227 LUCAS 2018 survey points are located within the EUSALP map extent. From these, a random selection of survey sites was made using the *subset feature* analysis tool in ArcGIS. The number of sites to be allocated to each LULC class was calculated as a function of their area proportion in the EUSALP map. In this way, the sampling design is not only systematic but also stratified. A minimum number of 20 sample units per LULC class was defined to ensure that even small strata were represented in the sample. However, for some strata there were no reference points available (41200, 42200). In the end, 2300 LUCAS 2018 points were randomly selected (see Figure S2).

An initial blind interpretation was performed, which consists in constructing the validation data without any knowledge of the map layer being evaluated. This was done by evaluating LULC on the reference points using EUSALPs’ LULC map classification codes. ESRI World Imagery (https://services.arcgisonline.com/ArcGIS/rest/services/World_Imagery/MapServer) and LUCAS 2018 thematic information were used for this first round of classification. As this method may underestimate the accuracy for complex and heterogeneous land use classes and potential land use changes (especially on arable land) or class definitions, we then used a plausibility approach, which is applied on all sample units that result in disagreement with the EUSALP LULC Map. This step consists in checking both classified values (blind validation and EUSALP map) for plausibility within the accepted product specifications, without knowing the corresponding classification source.

The overall map accuracy was assessed using an error matrix approach^35^. The producer accuracy (PA) and the user accuracy (UA) for each LULC class were evaluated in an area-weighted confusion matrix with 95% confidence interval. We obtained an overall accuracy (OA) of 88.8% ± 1.8 for the plausibility approach (Tables 4, 6, S3), which is a good result that meets validation standards, even though the blind evaluation showed substantially lower overall accuracy (64.8% ± 3.7) (Tables 5, S4).

**Table 4:** Plausibility evaluation: Estimated error matrix based on Table S3 with cell entries expressed as the estimated proportion of area (%).

Accuracy measures are presented with a 95% confidence interval.

**Table 5:** Blind evaluation: Estimated error matrix based on Table S4–expressed as the estimated proportion of area (%).

Accuracy measures are presented with a 95% confidence interval.

**Table 6:** Pixel count, total area, standard error of the adjusted area-estimate and 95% confidence interval for each acreage estimate of the EUSALP LULC Map classes.

Class	Pixels	Area [ha]	Std Error [ha]	95% Conf [ha]
Artificial surfaces and constructions	129,019,941	322,550	43,562	87,124
Dense settlement area (>30%)	185,039,653	462,599	42,333	84,666
Low density settlement area (<30%)	68,998,850	172,497	17,072	34,144
Builtup area	305,690,414	764,226	24,046	48,091
Open settlement area	102,764,003	256,910	16,193	32,385
Industrial and commercial zones	140,919,712	352,299	26,857	53,713
Road Networks & railways	590,250,386	1,475,626	33,776	67,551
Green urban areas	35,522,902	88,807	14,772	29,543
Cultivated areas - Arable Land	3,449,750,460	8,624,376	75,017	150,034
Permanent Crops	18,951,784	47,379	94,924	189,847
Vineyard	124,660,392	311,651	43,529	87,058
Orchard	87,646,510	219,116	35,522	71,045
Managed Grassland - Pastures -	1,390,491,303	3,476,228	195,749	391,497
Seminatural Grassland - Meadows	1,117,587,099	2,793,968	165,931	331,862
Broadleaf tree cover	33,480,573	83,701	19,191	38,383
Broadleaf tree cover 30–60%	534,125,818	1,335,315	52,541	105,082
Broadleaf tree cover 60–100%	3,163,093,328	7,907,733	99,273	198,546
Coniferous tree cover	22,657,922	56,645	30,836	61,673
Coniferous tree cover 30–60%	336,996,724	842,492	42,195	84,391
Coniferous tree cover 60–100%	3,101,178,390	7,752,946	23,678	47,355
Tree Cover	394,320,290	985,801	75,466	150,932
Tree cover in agricultural context	138,829,319	347,073	28,427	56,854
Tree cover in urban context	71,049,235	177,623	8,582	17,164
Green linear elements/woody features	255,049,543	637,624	55,269	110,538
Scrub and shrubland	802,900,990	2,007,252	120,658	241,317
Alpine and sub-alpine natural grassland	327,276,038	818,190	48,220	96,441
Moors and Heathland - other scrubland	7,953,134	19,883	3,667	7,334
Beaches, dunes, sands	13,165,630	32,914	14,302	28,604
Bare rocks and rock debris	209,975,282	524,938	97,129	194,257
Sparsely vegetated land	18,264,721	45,662	50,837	101,673
Permanent snow-covered surfaces	185,528,615	463,822	39,626	79,252
Wetland - inland marshes	41,487,179	103,718	22,943	45,886
Coastal salt marshes	4,886,556	12,216	21,817	43,633
Water bodies	257,221,913	643,055	31,876	63,752
River network	23,884,227	59,711	3,166	6,332
Lagoons and Estuaries	4,869,249	12,173	12,861	25,722

For easier interpretation, the road and railways, agricultural, green linear elements and river LULC classes were each aggregated into a single class.

For classes 41200, 42200, 52100 and 32200 there were too few sample points available. Therefore, these classes could not be properly validated^35^. However, this is of little concern as these LULC classes cover only 0.06% of the total map area. Only 17 reference points could not be classified.

The OA of the EUSALP LULC map is very similar to the OA of the various input datasets and it would be very unlikely that the output is better than the input. Therefore, we are confident that the map creation approach was successful and that the created dataset meets accuracy standards.

Insight into the temporal extent of the LULC data is given by using the EUSALP_LULC_data_sources.tif raster^31^, which shows the reference year of each map cell. Information on the reference year exists for each input data layers except for Open Street Map.

Logical and format consistency of our map is ensured by the harmonization steps each data input file has undergone (the MMU is pixel based, the Coordinate Reference System is set to EPSG 3035, Pixel size is set to 5 m). Overlap cannot occur due to the final data format.

Positional accuracy could not be assessed due to missing reference data with sufficient spatial accuracy. However, all of the input data used have been evaluated for positional accuracy during the validation process.

## Usage Notes

The EUSALP LULC map has a high potential for customization as the regrouping of the 65 LULC classes allows for interest-specific reclassifications in any GIS program. Due to the high level of detail, our map can be used even at the local scale, having a level of detail near artificial structures and settlements comparable to maps at 1:5,000 scale.

**Fig. 3:**
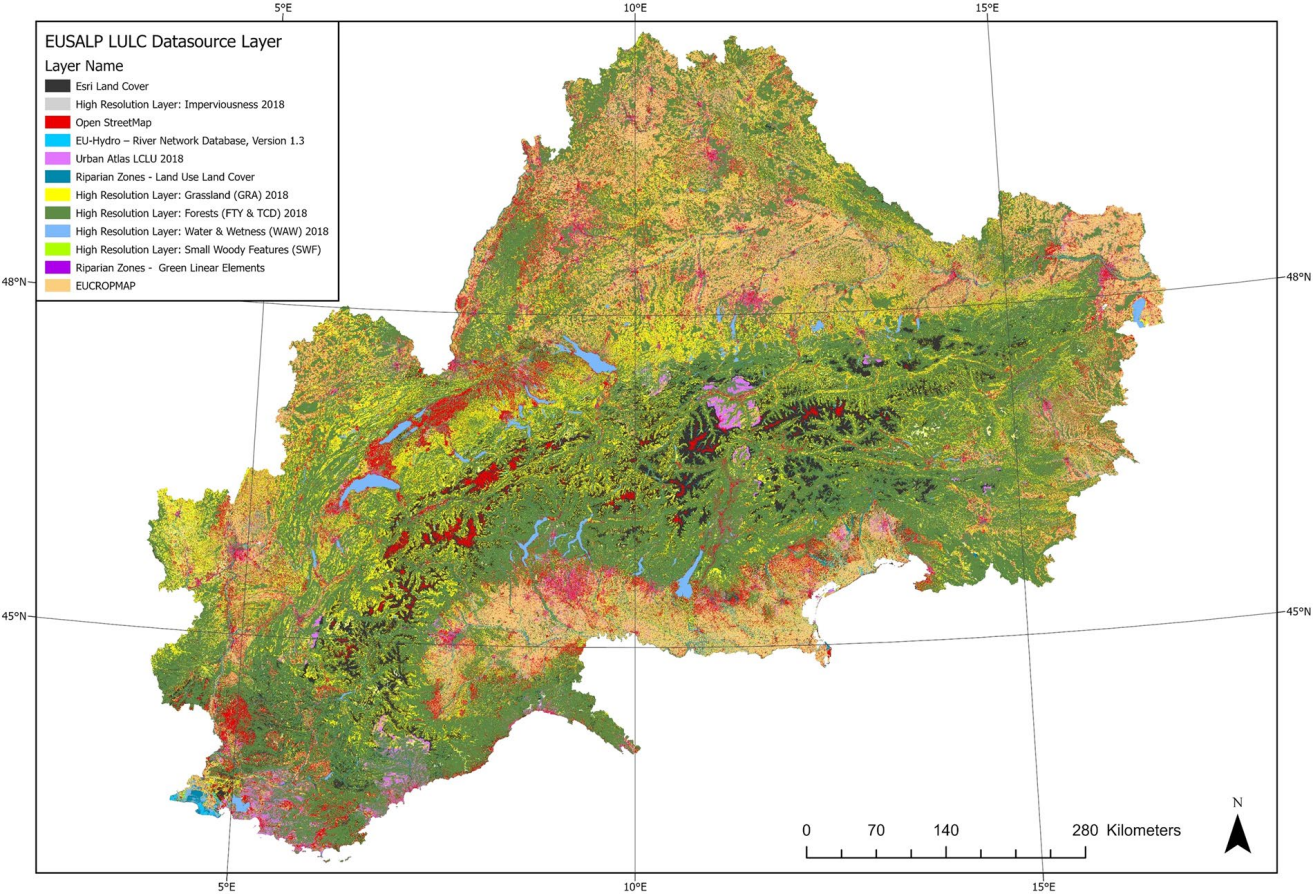
Map of the Datasource Layer which indicates the source and reference year of every pixel.

However, the EUSALP LULC map still holds some limits and improvement potential. Indeed, the time dimension of different data layers needs to be carefully considered when using the map: in fact, although corresponding to the newest available high-resolution data layers, the combined data are from different years. If time specificity is required, the user needs to refer to the Datasource layer (Fig. 3).

## Supplementary information

SUPPLEMENTARY INFORMATION

## Data Availability

No custom code has been used during the generation and processing of this dataset.
