# Supplementary material for: A detailed land use/land cover map for the European Alps macro region
Source: Sci Data. 2023 Jul 19;10:468. doi: 10.1038/s41597-023-02344-3 (PMC10356817; doi:10.1038/s41597-023-02344-3)
Supplement: Supplementary file 1 — SUPPLEMENTARY INFORMATION [file 41597_2023_2344_MOESM1_ESM.docx]

Supplementary Materials to

**A detailed land use/land cover map for the European Alps macro region**

| 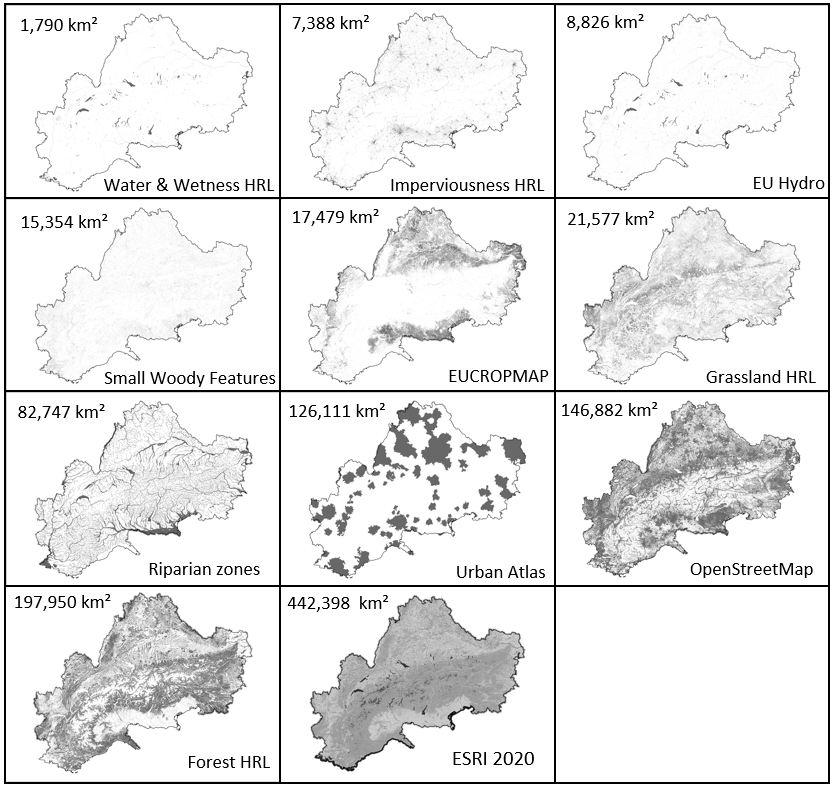 |
| --- |
| **Figure S1:**  Area covered by the single Input datasets. Due to overlap, not the entire area of each dataset is present in the final map. |

| 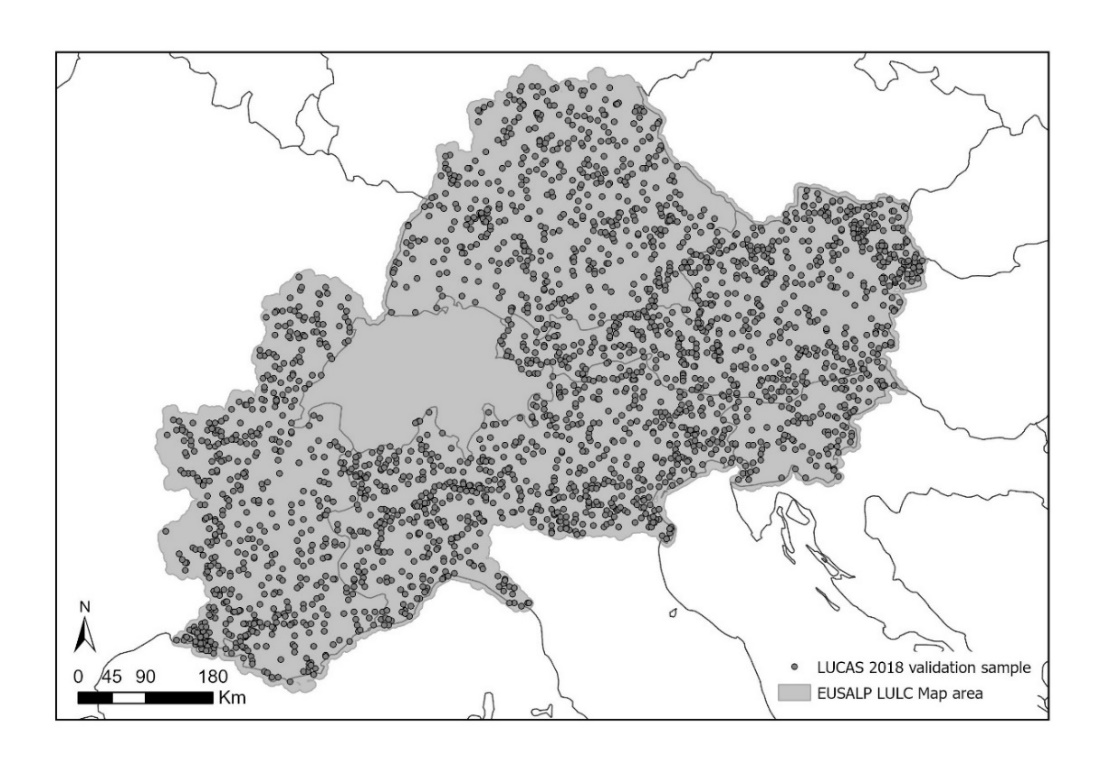 |
| --- |
| **Figure S2:** Distribution of selected LUCAS 2018 survey points for validation dataset |

| **Table S1**: List of selected OSM features (number of features included in the map: Roads 6,901,902; Landuse 2,823,422; Buildings 38,354,584) |
| --- |
| \| **Dataset** \| **OSM_key** \| **Feature type** \| **Buffer [m]** \| \| --- \| --- \| --- \| --- \| \| OSM_landuse \| farmland \| polygon \| - \| \| farmyard \| polygon \| - \| \| grass \| polygon \| - \| \| meadow \| polygon \| - \| \| orchard \| polygon \| - \| \| vineyard \| polygon \| - \| \| commercial \| polygon \| - \| \| industrial \| polygon \| - \| \| retail \| polygon \| - \| \| cemetery \| polygon \| - \| \| park \| polygon \| - \| \| recreation_ground \| polygon \| - \| \| cycleway \| polygon \| - \| \| living_street \| polygon \| - \| \| OSM_natural \| glacier \| polygon \| - \| \| OSM_buildings \| buildings \| polygon \| - \| \| OSM_roads \| motorway \| polyline \| 11 \| \| motorway_link \| polyline \| 11 \| \| primary \| polyline \| 6 \| \| primary_link \| polyline \| 6 \| \| residential \| polyline \| 3 \| \| secondary \| polyline \| 6 \| \| secondary_link \| polyline \| 6 \| \| service \| polyline \| 3 \| \| tertiary \| polyline \| 3 \| \| tertiary_link \| polyline \| 3 \| \| trunk \| polyline \| 3 \| \| trunk_link \| polyline \| 3 \| \| unclassified \| polyline \| 3 \| \| unknown \| polyline \| 3 \| \| OSM_railways \| light_rail \| polyline \| 3 \| \| monorail \| polyline \| 3 \| \| narrow_gauge \| polyline \| 3 \| \| rail \| polyline \| 6 \| \| tram \| polyline \| 3 \| |

| **Table S2**: LULC datasets used to build the EUSALP map with links to validation reports and Terms of use |
| --- |
| \| **Source** \| **Validation Report** \| **Terms of use** \| \| --- \| --- \| --- \| \| ESRI 2020 Land Cover Map \| <https://ieeexplore.ieee.org/document/9553499> \| [Creative Commons Attribution 4.0 International (CC BY 4.0) licence](https://creativecommons.org/licenses/by/4.0/) \| \| Imperviousness high resolution layer (HRL) \| <https://land.copernicus.eu/user-corner/technical-library/clms_hrl_imd_validation_report_sc04_1_3.pdf> \| [open and free access - Copernicus data and information policy Regulation (EU) No 1159/2013 of 12 July 2013](https://eur-lex.europa.eu/legal-content/EN/TXT/?uri=CELEX%3A32013R1159) \| \| Grassland HRL \| <https://land.copernicus.eu/user-corner/technical-library/clms_hrl_gra_validation_report_sc04_v1_5.pdf> \| [open and free access - Copernicus data and information policy Regulation (EU) No 1159/2013 of 12 July 2013](https://eur-lex.europa.eu/legal-content/EN/TXT/?uri=CELEX%3A32013R1159) \| \| Forest HRL \| <https://land.copernicus.eu/user-corner/technical-library/comparative-validation> \| [open and free access - Copernicus data and information policy Regulation (EU) No 1159/2013 of 12 July 2013](https://eur-lex.europa.eu/legal-content/EN/TXT/?uri=CELEX%3A32013R1159) \| \| Water and Wetness HRL \| <https://land.copernicus.eu/user-corner/technical-library/water-wetness-2018-user-manual.pdf> \| [open and free access - Copernicus data and information policy Regulation (EU) No 1159/2013 of 12 July 2013](https://eur-lex.europa.eu/legal-content/EN/TXT/?uri=CELEX%3A32013R1159) \| \| EUCROPMAP \| <https://ars.els-cdn.com/content/image/1-s2.0-S0034425721004284-mmc1.pdf>  <https://ieeexplore.ieee.org/document/9553758> \| [Creative Commons Attribution 4.0 International (CC BY 4.0) licence](https://creativecommons.org/licenses/by/4.0/) \| \| *OpenStreetMap (OSM) \| <https://wiki.openstreetmap.org/wiki/Tasking_Manager/Validating_data> \| [Creative Commons Attribution-ShareAlike 2.0 (CC BY-SA 2.0)](https://creativecommons.org/licenses/by-sa/2.0/) \| \| Urban Atlas 2018 \| <https://land.copernicus.eu/user-corner/technical-library/urban-atlas-2018-validation-report> \| [open and free access - Copernicus data and information policy Regulation (EU) No 1159/2013 of 12 July 2013](https://eur-lex.europa.eu/legal-content/EN/TXT/?uri=CELEX%3A32013R1159) \| \| Small Woody Features HRL \| <https://land.copernicus.eu/user-corner/technical-library/hrl-small-woody-features-2015-validation-report> \| [open and free access - Copernicus data and information policy Regulation (EU) No 1159/2013 of 12 July 2013](https://eur-lex.europa.eu/legal-content/EN/TXT/?uri=CELEX%3A32013R1159) \| \| Landuse Riparian Zone & Green linear elements \| <https://land.copernicus.eu/user-corner/technical-library/riparian_zones_validation_report_2018_partial> \| [open and free access - Copernicus data and information policy Regulation (EU) No 1159/2013 of 12 July 2013](https://eur-lex.europa.eu/legal-content/EN/TXT/?uri=CELEX%3A32013R1159) \| \| **EU Hydro - Rivers and Inland water \| <https://land.copernicus.eu/user-corner/technical-library/eu-hydro-validation-report> \| [open and free access - Copernicus data and information policy Regulation (EU) No 1159/2013 of 12 July 2013](https://eur-lex.europa.eu/legal-content/EN/TXT/?uri=CELEX%3A32013R1159) \| |
|  |

| **Table S3:** Error matrix of sample count of Plausibility evaluation |
| --- |
| **** |

| **Table S4** Error matrix of sample count of Blind evaluation |
| --- |
| **** |

**Table S5**: EUSALP Landuse classes data origin.

| Code | EUSALP Code | ESRI Landcover | Urban Atlas | OSM | Riparian Zones | High Resolution Layers | EUCROPMAP |
| --- | --- | --- | --- | --- | --- | --- | --- |
| 11000 | Artificial surfaces and constructions | Built Area | Port areas | farmyard | Greenhouses | IMD >50% |  |
|  |  |  | Airport |  | Transport infrastructure |  |  |
|  |  |  | Mineral Extraction |  |  |  |  |
|  |  |  | Construction sites |  |  |  |  |
|  |  |  | Land without current use |  |  |  |  |
| 11100 | Dense settlement area (>30%) |  | Continuous Urban Fabric >80% |  | Dense Urban Fabric 30-80% |  |  |
|  |  |  | Discontinuous **dense urban fabric** (S.L:50% - 80%) |  |  |  |  |
| 11200 | Low density settlement area (<30%) |  | Discontinuous very low density urban fabric (S.L:<10%) |  | Low Density <30% |  |  |
| 11300 | Builtup area |  |  | Buildings |  | IBU = 1 |  |
| 11400 | Open settlement area |  |  | Delineate Buitup area 100m Scale 1:10000 |  |  |  |
| 12100 | Industrial and commercial zones |  | Industrial, commercial, public, military and private units |  | Industrial, Commercial |  |  |
| 12210 | Roads motorways and trunks |  | Fast transit roads and associated land | Roads motroways and trunks |  |  |  |
| 12220 | Road Networks |  | Other roads and associated land |  | Road Networks |  |  |
| 12221 | Roads tertiary and others |  |  | Roads tertiary and others |  |  |  |
| 12230 | Railways train tracks |  | Railways and associated land | Railway | Railways |  |  |
| 12240 | Unpaved Roads and Tracks |  |  | Tracks Grade1-5 |  |  |  |
| 14100 | Green urban areas |  | Green urban areas | Park | Green urban |  |  |
|  |  |  | Sports and leisure facilities | recreation ground |  |  |  |
|  |  |  |  | cemetery |  |  |  |
| 21000 | Cultivated areas - Arable Land - Annual Crops | Crops | Arable Land |  | Annual crops  Land pricipally agriculture | Ploughed Fields |  |
| 21211 | Common wheat |  |  |  |  |  | Common wheat |
| 21212 | Durum wheat |  |  |  |  |  | Durum wheat |
| 21213 | Barley |  |  |  |  |  | Barley |
| 21214 | Rye |  |  |  |  |  | Rye |
| 21215 | Oats |  |  |  |  |  | Oats |
| 21216 | Maize |  |  |  |  |  | Maize |
| 21217 | Rice |  |  |  |  |  | Rice |
| 21218 | Triticale |  |  |  |  |  | Triticale |
| 21219 | Other cereals |  |  |  |  |  | Other cereals |
| 21221 | Potatoes |  |  |  |  |  | Potatoes |
| 21222 | Sugar beet |  |  |  |  |  | Sugar beet |
| 21223 | Other root crops |  |  |  |  |  | Other root crops |
| 21230 | Other non permanent industrial crops |  |  |  |  |  | Other non permanent industrial crops |
| 21231 | Sunflower |  |  |  |  |  | Sunflower |
| 21232 | Rape and turnip rape |  |  |  |  |  | Rape and turnip rape |
| 21233 | Soya |  |  |  |  |  | Soya |
| 21240 | Dry pulses |  |  |  |  |  | Dry pulses |
| 21250 | Fodder crops (cereals and leguminous) |  |  |  |  |  | Fodder crops (cereals and leguminous) |
| 21290 | Bare arable land |  |  |  |  |  | Bare arable land |
| 22000 | Permanent Crops |  | Permanent Crops |  | Olive groves |  |  |
|  |  |  |  |  | Agro-forestry |  |  |
| 22100 | Vinyard |  |  | Vinyard |  |  |  |
| 22200 | Orchard |  |  | Orchard |  |  |  |
| 23100 | Managed Grassland - Pastures - | Grassland < 1600m, <26° | Herbaceous vegetation associations (natural grassland, moors...) |  | Managed grassland | Grassland < 1600m, <26°slope |  |
| 23200 | Seminatural Grassland - Meadows | Grassland < 2000m, >26° | Herbaceous vegetation associations (natural grassland, moors...) | Meadows | Semi-natural grassland | Grassland > 1600m < 2000m, >26°slope |  |
| 31100 | Broadleaf tree cover |  |  |  | Natural and seminatural Broadleaved forest |  |  |
|  |  |  |  |  | Highly artificial Broadleaved forest |  |  |
| 31102 | Broadleaf tree cover 30-60% |  |  |  |  | FTY = 1 & TCD 30-60% |  |
| 31103 | Broadleaf tree cover 60-100% |  |  |  |  | FTY = 1 & TCD >60% |  |
| 31200 | Coniferous tree cover |  |  |  | Natural and seminatural Coniferous forest |  |  |
|  |  |  |  |  | Highly artificial Coniferous forest |  |  |
| 31202 | Coniferous tree cover 30-60% |  |  |  |  | FTY = 2 & TCD 30-60% |  |
| 31203 | Coniferous tree cover 60-100% |  |  |  |  | FTY = 2 & TCD >60% |  |
| 31300 | Tree Cover | Trees |  |  | Woodland and forest |  |  |
|  |  |  |  |  | Natural and seminatural Mixed forest |  |  |
|  |  |  |  |  | Highly artificial Mixed forest |  |  |
| 31400 | Other tree cover - agricultural |  |  |  |  | FADSL = 3 |  |
| 31450 | Other tree cover - urban |  |  |  |  | FADSL = 4, 5 |  |
| 31500 | Green Linear Elements - linear Woody Features |  |  |  | Green Linear Elements | Small Woody Features - Linear |  |
|  |  |  |  |  | Lines of trees and scrub |  |  |
| 31600 | patchy woody features |  |  |  |  | Patchy woody features |  |
| 31610 | Additional Woody Features |  |  |  |  | Additional Woody Features |  |
| 32000 | Scrub/Shrub | Scrub/Shrub |  |  |  |  |  |
| 32100 | Alpine and sub-alpine natural grassland |  | Herbaceous vegetation associations (natural grassland, moors...) > 2000m |  | Alpine and sub-alpine natural grassland > 2000m | Grassland >2000m |  |
| 32200 | Moors and Heathland - other scrubland |  |  |  | Heathland and scrub |  |  |
|  |  |  |  |  | Moors and heathland |  |  |
|  |  |  |  |  | Heathland and Moorland |  |  |
|  |  |  |  |  | Other scrub land |  |  |
| 32300 | Sclerophyllous vegetation |  |  |  | Sclerophyllous vegetation |  |  |
| 33000 | Bare Ground | Bare Ground |  |  |  |  |  |
| 33100 | Beaches, dunes, sands |  |  |  | Beaches, dunes, sands |  |  |
| 33200 | Bare rocks and rock debris |  |  |  | Bare rocks and rock debris |  |  |
| 33300 | Sparsely vegetated land |  | Open spaces with little or no vegetation |  | Transitional woodland scrub |  |  |
|  |  |  |  |  | Burnt areas |  |  |
|  |  |  |  |  | Damaged forest |  |  |
| 33500 | Permanent snow covered surfaces | Snow Ice |  | Glacier | Glaciers and perpetual snow |  |  |
| 41000 | Wetland (permanent wet areas) - inland marshes | Flooded Vegetation | Wetland |  | Wetland | WAW = 3 |  |
|  |  |  |  |  | Inland marshes |  |  |
| 41200 | Peatbogs |  |  |  | Exploited peat bog |  |  |
|  |  |  |  |  | Unexploited peat bog |  |  |
| 42100 | Coastal salt marshes |  |  |  | Coastal salt marshes |  |  |
|  |  |  |  |  | Salines |  |  |
| 42200 | Intertidal flats |  |  |  | Intertidal flats |  |  |
| 51000 | Water bodies | Water | Water |  |  | WAW = 1 |  |
| 51100 | Rivernetwork < 10m |  |  |  |  | EU Hydro |  |
| 51200 | Riverbed > 10m width |  |  |  |  | EU Hydro |  |
| 52100 | Lagoons and Estuaries |  |  | Coastal lagoons |  |  |  |
|  |  |  |  | Estuaries |  |  |  |
